# Cyclic nucleotide permeability through unopposed connexin hemichannels

**DOI:** 10.3389/fphar.2013.00075

**Published:** 2013-06-06

**Authors:** Virginijus Valiunas

**Affiliations:** Department of Physiology and Biophysics, Stony Brook UniversityStony Brook, NY, USA

**Keywords:** connexin43, connexin26, electrophysiology, gap junction, permeability, cyclic AMP

## Abstract

Cyclic adenosine monophosphate (cAMP) is a well-known intracellular and intercellular second messenger. The membrane permeability of such molecules has potential importance for autocrine-like or paracrine-like delivery. Here experiments have been designed to demonstrate whether gap junction hemichannels, composed of connexins, are a possible entrance pathway for cyclic nucleotides into the interior of cells. HeLa cells stably expressing connexin43 (Cx43) and connexin26 (Cx26) were used to study the cyclic nucleotide permeability of gap junction hemichannels. For the detection of cAMP uptake, the cells were transfected using the cyclic nucleotide-modulated channel from sea urchin sperm (SpIH) as the cAMP sensor. SpIH derived currents (*I*_m_) were recorded in whole-cell/perforated patch clamp configuration. Perfusion of the cells in an external K^+^ aspartate^-^ (KAsp) solution containing 500 μM cAMP and no extracellular Ca^2^^+^, yielded a five to sevenfold increase in the *I*_m_ current level. The SpIH current increase was associated with detectable hemichannel current activity. Depolarization of cells in Ca^2^^+^-free NaCl perfusate with 500 μM cAMP also induced a SpIH current increase. Elevating extracellular Ca^2^^+^ to mM levels inhibited hemichannel activity. Perfusion with a depolarizing KAsp solution containing 500 μM cAMP and 2 mM Ca^2^^+^ did not increase SpIH currents. The addition of the gap junction blocker carbenoxolone to the external solution inhibited cAMP uptake. Both cell depolarization and lowered extracellular Ca^2^^+^ increase the open probability of non-junctional hemichannels. Accordingly, the SpIH current augmentation was induced by the uptake of extracellular cAMP via open membrane hemichannels in Cx43 and Cx26 expressing cells. The data presented here show that hemichannels of Cx43 and Cx26 are permeable to cAMP, and further the data suggest that hemichannels are, in fact, a potential pathway for cAMP mediated cell-to-cell signaling.

## INTRODUCTION

Hemichannels or connexons are composed of connexins, which are synthesized in the endoplasmic reticulum and assembled into hexameric structures in the Golgi, and subsequently delivered to the plasma membrane via transport vesicles (Segretain and Falk, 2004). Hemichannels are large conductance membrane channels that for many years were thought to be silent or nearly silent subunits. Their function was thought to be only when two such channels, between closely apposed cells, formed an intercellular channel, the gap junction, containing an aqueous pore exclusive of the extracellular space. Once formed, gap junction channels eventually coalesce into aggregates or plaques consisting of hundreds to thousands of channels (Maurer and Weingart, 1987;Bukauskas et al., 2000). However, not all hemichannels are destined to become component parts of a gap junction channel. Rather, many are apparently randomly distributed within the plasma membrane. The presence of hemichannels within plasma membranes has been well-documented *in vitro* (De and Schwartz, 1992;Trexler et al., 1996;Valiunas and Weingart, 2000;Valiunas, 2002) and has prompted speculation about their role in cellular processes like volume regulation (Quist et al., 2000), the influx/efflux of metabolically relevant solutes such as ATP (Bruzzone et al., 2001;Dahl and Locovei, 2006), and cell death (Plotkin et al., 2002;Kalvelyte et al., 2003). The electrophysiological data collected in vitro have demonstrated that hemichannels open probability is increased with membrane depolarization in the presence of lowered extracellular calcium. The open probability can be reduced by acidic pH, calcium, trivalent cations, and quinine derivatives (Trexler et al., 1999;Contreras et al., 2002;Eskandari et al., 2002;Stout et al., 2002;Valiunas, 2002;Srinivas et al., 2005).

Cell to cell communication mediated by gap junction channels represents one of two established intercellular pathways for the movement of signaling molecules, metabolites, and siRNA/miRNA from one cell to another (Valiunas et al., 2005;Kanaporis et al., 2008). Exocytosis/endocytosis is another intercellular delivery pathway that utilizes the extracellular volume for autocrine and paracrine mediated signaling. The suggested possible roles for hemichannels, such as volume regulation and/or cell death would also be examples where delivery is via the extracellular space and hence, represents an example of autocrine/paracrine-like delivery. Furthermore, a recent review byWang et al. (2013) also suggests hemichannels are a significant source of autocrine and paracrine messengers. When considering the role of hemichannels in such a delivery system it is best to consider the delivery pathway as autocrine-like or paracrine-like because delivery of a solute does not necessarily involve vesicular traffic nor is it necessarily mediated by surface receptors.

Inevitably, an interesting question arises: are hemichannels an alternate autocrine/paracrine-like pathway for delivery of relevant signaling molecules, like adenosine and other related compounds? Two examples focus on cyclic adenosine monophosphate (cAMP) as a signal molecule candidate. The extracellular release of cAMP is known to exert effects such as receptor expression in renal cells (Kuzhikandathil et al., 2011) and inhibition of skeletal muscle inotropism (Duarte et al., 2012). Before addressing further questions, such as if hemichannels are involved in a paracrine-like delivery of cAMP, it is essential to understand the characteristics of hemichannel permeability.

As a first step in assessing hemichannels as a potential delivery pathway, HeLa cells were transfected with cyclic nucleotide sensor SpIH in order to investigate the permeability of cAMP of unopposed connexin43 (Cx43) and connexin26 (Cx26) hemichannels.

## MATERIALS AND METHODS

### CELLS AND CULTURE CONDITIONS

Experiments were carried out on human HeLa cells stably transfected with wild-type mCx43 and hCx26. HeLa cells were grown in Dulbecco’s Modified Eagle Medium (DMEM; Gibco BRL), supplemented with 10% fetal calf serum (FCS; Hyclone), 100 mg/mL streptomycin (Gibco BRL), and 100 U/mL penicillin (Gibco BRL). The medium also contained 100 mg/mL hygromycin (Sigma) or 1 mg/mL puromycin (Sigma). The cells were passaged weekly, diluted 1:10, and kept at 37°C in a CO_2_ incubator (5% CO_2_/95% ambient air). Culture conditions for these cells have been previously published in complete detail (Valiunas et al., 2000, 2001). Electrophysiological experiments were carried out on single cells cultured for 1–3 days.

### IMMUNOFLUORESCENT LABELING OF CONNEXINS

HeLa cells expressing Cx26 and/or Cx43 were grown on coverslips and stained as described earlier (Laing and Beyer, 1995). Commercially available anti-connexin43 (Sigma) and anti-connexin26 (Zymed Labs) antibodies were used for immunostaining. Alexa Fluor488 conjugated anti-rabbit IgG (Cell Signaling) was used as a secondary antibody. The protein expression and localization was monitored with a 63× oil objective on a Zeiss Axiovert 200 inverted microscope and Axiovision (Zeiss) software.

### ELECTROPHYSIOLOGICAL MEASUREMENTS

Experiments were carried out on single cells using the whole-cell/perforated patch voltage-clamp technique to control the membrane potential and to measure membrane currents of the cell. For electrical recordings, glass coverslips with adherent cells were transferred to an experimental chamber mounted on the stage of an inverted microscope (Olympus- IX71). During experiments, the cells were superfused with depolarizing bath solution (KAsp) at room temperature (~22°C) containing (mM): K^+^ aspartate^-^ 120; NaCl 10; CaCl_2_ 2; HEPES 5 (pH 7.4); glucose 5; 2 mM CsCl, BaCl_2_ and TEA^+^ Cl^-^ were also added. For the Ca^2^^+^-free (0 Ca^2^^+^) bath solution CaCl_2_ was omitted. For the regular modified Tyrode external solution (NaCl), K^+^ aspartate^-^ in the superfusate was replaced with an equal molar concentration of NaCl. The patch pipettes were filled with solution containing (mM): K^+^ aspartate^-^, 120; NaCl, 10; MgATP, 3; HEPES, 5 (pH 7.2); EGTA, 10 (pCa ~8); filtered through 0.22 μm pores. In perforated patch experiments, the pipette solution contained 30–50 μM β-escin (Fan and Palade, 1998). The series resistance with β-escin patches measured 11–20 MΩ. Patch pipettes were pulled from glass capillaries (code GC150F; Harvard Apparatus) with a horizontal puller (DMZ-Universal, Zeitz-Instrumente). When filled, the resistance of the pipettes measured 1–4 MΩ.

### cAMP-UPTAKE STUDIES

Cyclic AMP transfer through gap junction hemichannels was investigated using single HeLaCx43 and/or HeLaCx26 cells. For the detection of cAMP uptake, the cells were transfected with the cAMP sensor, a cyclic nucleotide-modulated channel from sea urchin sperm (SpIH;Gauss et al., 1998;Shin et al., 2001;Kanaporis et al., 2008). Production, characterization, culture conditions, staining, and visualization of these cells have been described previously inKanaporis et al. (2008).

Wild-type, Cx43 and Cx26 transfected cells were incubated in either in NaCl or K^+^ aspartate^-^ (KAsp) bath solution (with 2 mM Ca^2^^+^ or Ca^2^^+^-free). For cAMP uptake experiments cAMP (Sigma-Aldrich) was added to the external bath solution to reach a concentration of 500 μM. The SpIH derived currents (*I*_m_) were recorded from the single cell expressing SpIH and Cx43 and/or Cx26. In some experiments 50 μM of cAMP was introduced via the patch pipette directly in to the cell. In another series of experiments 500 μM cAMP was also locally introduced to the cell membrane via the external pipette. To prevent cAMP degradation a membrane-permeable phosphodiesterase inhibitor IBMX (200 mM, Sigma-Aldrich) was added to the bath solution. An adenylate cyclase inhibitor, 2′,5′-dideoxyadenosine (5 mM, Calbiochem) was added to the pipette and bath solutions to inhibit intracellular cAMP production.

### SIGNAL RECORDING AND ANALYSIS

Voltage and current signals were recorded using patch clamp amplifiers (Axopatch 200). The current signals were digitized with a 16 bit A/D-converter (Digidata 1322A; Molecular Devices) and stored within a personal computer. Data acquisition and analysis were performed with pClamp9 software (Molecular Devices). Statistical analysis was performed using SigmaStat (Jandel Scientific). The Mann–Whitney Rank Sum test was used for all cases unless otherwise noted. The results are presented as means ± SEM.

## RESULTS

### LOCALIZATION OF CONNEXINS WITHIN CELLS

**FIGURE 1:**
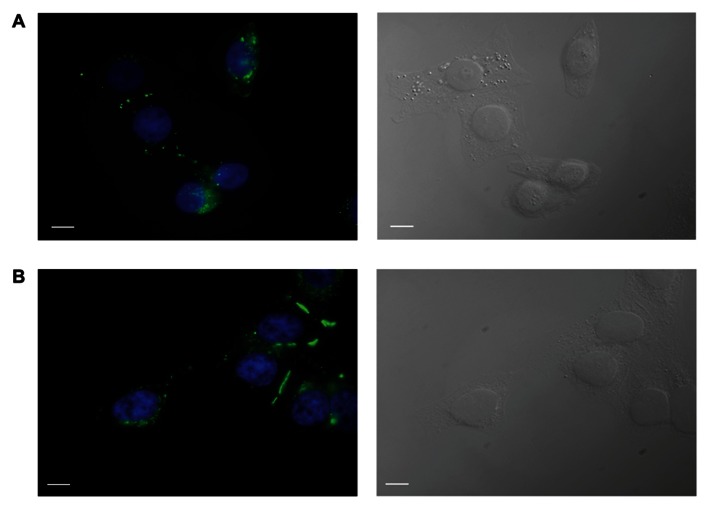
**Immunofluorescent identification of connexin expression in transfected HeLa cells.** HeLa cells expressing Cx43 **(A)** and Cx26 **(B)** stained with antibodies to Cx43 and Cx26, respectively, show typical punctate staining of Cx43 and Cx26 gap junction plaques at cell–cell contact areas, as well as Cx43 and Cx26 localization in the cell membranes of single cells. Green labeling represents staining of connexin proteins, while blue shows DAPI staining of cell nuclei. The right panels show bright field images. Scale bar, 10 μm.

HeLa cells stably transfected with mCx43 and hCx26 were immunostained with anti-Cx43 and anti-Cx26 antibodies, respectively. Immunofluorescent staining verified protein expression and localization of Cx43 and Cx26 within the cells (**Figure 1). As shown in Figure 1**, typical punctate staining (in green) at the cell to cell contact regions and the cell membranes of single cells indicates Cx43 (A) and Cx26 (B) expression in HeLa cells stably transfected with Cx43 and Cx26, respectively.

### PROPERTIES OF cAMP REPORTER CURRENTS

For detecting extracellular cAMP permeation through membrane hemichannels, HeLa cells expressing Cx43 or Cx26 were transfected with the cyclic nucleotide-modulated channel from sea urchin sperm (SpIH;Gauss et al., 1998;Shin et al., 2001) as a cAMP sensor. The activity of SpIH channels in transfected cells was determined using a single cell whole-cell perforated patch clamp. It has been shown that intracellular perfusion with cAMP increased SpIH currents by more than fivefold in a dose-dependent manner (Kanaporis et al., 2008). The cAMP dose-response curve and characterization of SpIH channels derived currents were reported in further detail earlier inKanaporis et al. (2008).

**FIGURE 2:**
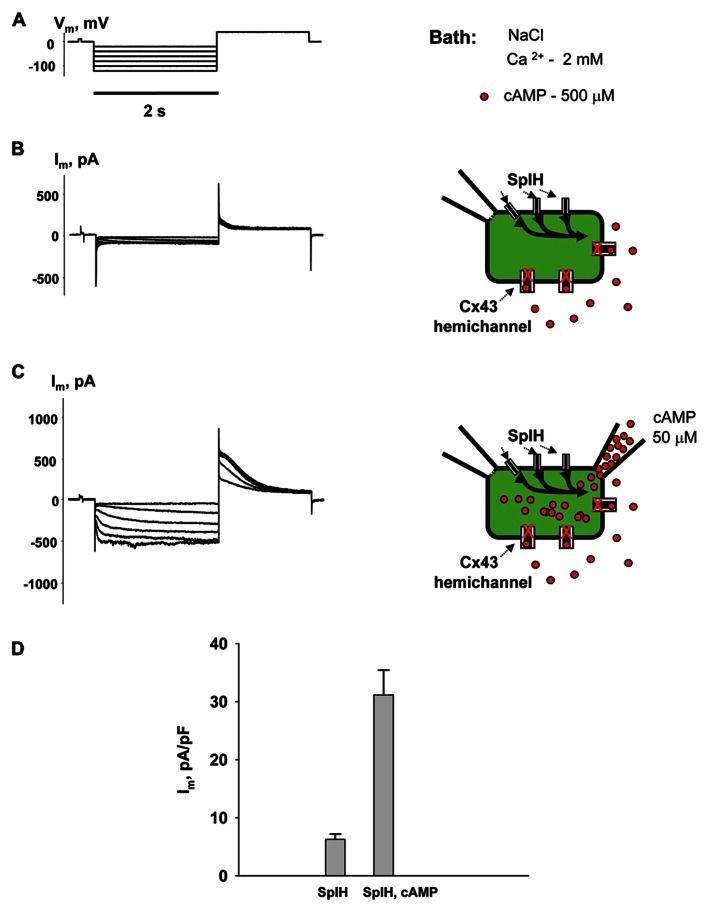
**Properties of SpIH channels.** **(A)** Voltage protocol (*V*_m_) and whole-cell currents (*I*_m_) recorded in SpIH transfected HeLaCx43 cells in the absence **(B)** and presence **(C)** of 50 μM cAMP in the pipette solution. Schematics in the right panels illustrate whole-cell recording conditions from HeLaCx43/SpIH cells. The NaCl external bath solution contained 2 mM Ca^2^^+^ and 500 μM cAMP. **(D)** Average of tail current densities measured after voltage step to *V*_m_ = -100 mV in the absence (6.2 ± 0.9 pA/pF, *n* = 10 cells) and the presence of intracellular cAMP (31.4 ± 4.3 pA/pF, *n* = 8 cells), *P* < 0.001.

**Figure 2** shows the voltage protocol (*V*_m_) (A) along with the two resultant membrane currents (*I*_m_) recorded from the same HeLaCx43 cell transfected with SpIH (B and C). The cell was perfused with the external NaCl (modified Tyrode) solution containing 2 mM Ca^2^^+^ and 500 μM cAMP. **Figure 2B** (left panel) shows whole-cell (perforated patch) currents recorded from a SpIH transfected cell, while the right panel demonstrates experiment conditions. **Figure 2C** (left panel) shows the current recording from the same cell after the second pipette with 50 μM cAMP in whole-cell mode was attached to it (shown in the right panel). Voltage pulses delivered from a holding potential of 0 mV to test potentials between -20 and -120 mV produced time- and voltage-dependent inward and tail currents (*V*_m_ = +50 mV) in SpIH transfected cells. When cAMP was present in the pipette solution, SpIH transfected cells exhibited larger currents. The tail current densities were measured after voltage step to *V*_m_ = -100 mV. On average, intracellular application of cAMP increased the peak current level ~fivefold, in comparison to the SpIH transfected cells not treated with cAMP (31.4 ± 4.3 versus 6.2 ± 0.9 pA/pF, **Figure 2D**). These results are consistent with those found in a previous report (Kanaporis et al., 2008).

### cAMP TRANSFER THROUGH MEMBRANE HEMICHANNELS: MODULATION BY EXTERNAL Ca^2^^+^

**FIGURE 3:**
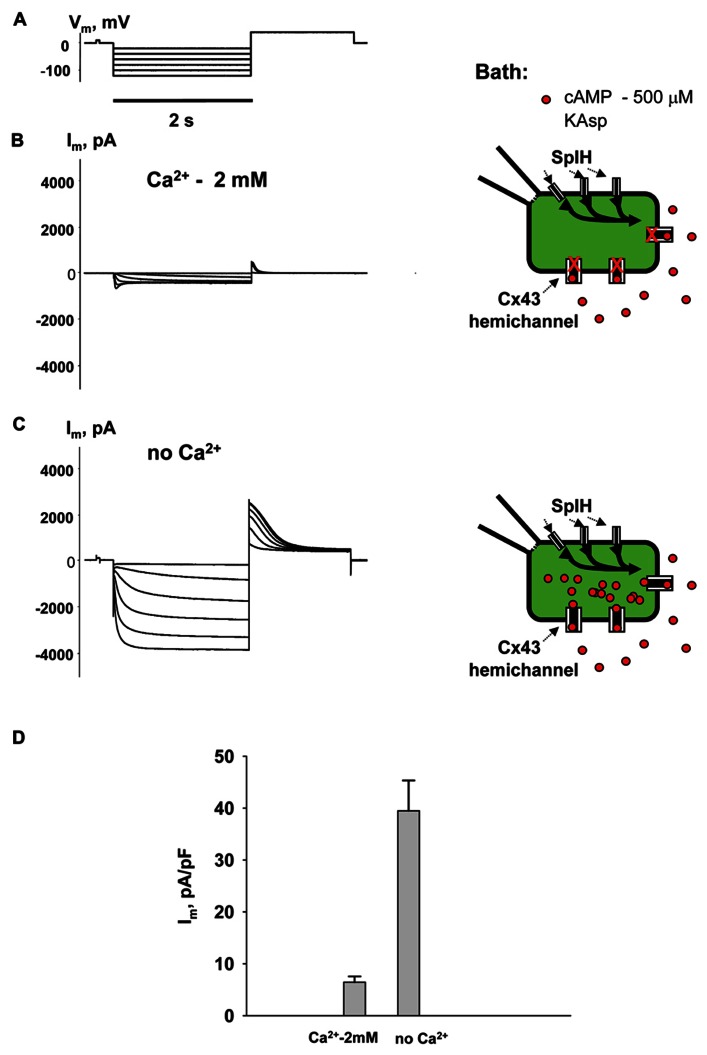
**cAMP induced activation of SpIH channels.** *I*_m_ elicited by hyperpolarizing pulses **(A)** (from -20 to -120 mV) in SpIH transfected HeLaCx43 cells in with both 2 mM Ca^2^^+^ **(B)** and with no added Ca^2^^+^ **(C)**. Schematics in the right panels of **(B)** and **(C)** illustrate the experimental conditions. In both cases the external KAsp bath solution contained 500 μM cAMP. *I*_m_ increased significantly with *V*_m_ and hyperpolarization induced voltage- and time-dependent inward currents when no external Ca^2^^+^ was present. **(D)** Average of current amplitudes recorded in the presence and in the absence of external Ca^2^^+^, respectively: 6.4 ± 1.1 pA/pF, *n* = 10 versus 39.5 ± 5.8 pA/pF, *n* = 13; *P* < 0.001.

To examine cAMP permeation through hemichannels within the plasma membrane SpIH transfected HeLa Cx43 and Cx26 cells were incubated in bath solutions (with and without 2 mM Ca^2^^+^) containing 500 μM cAMP. The left hand panels in **Figure 3B** show the recorded response of SpIH whole-cell (perforated patch) currents to hyperpolarizing voltage (*V*_m_; **Figure 3A**). The recordings were obtained from HeLaCx43 cell perfused with KAsp bath solution with 2 mM Ca^2^^+^. On average, *I*_m_ measured from 10 preparations yielded 6.4 ± 1.1 pA/pF. **Figure 3C** (left panel) illustrates that the SpIH current increased significantly (~sixfold) in the absence of external Ca^2^^+^ (39.5 ± 5.8 pA/pF, *n* = 13). The current amplitudes recorded in the presence and absence of external Ca^2^^+^ are summarized in **Figure 3D**. The SpIH current increase is consistent with the opening of Cx43 hemichannels in Ca^2^^+^-free solution and cAMP flux into the cell following the activation of SpIH channels (Contreras et al., 2003a).

**FIGURE 4:**
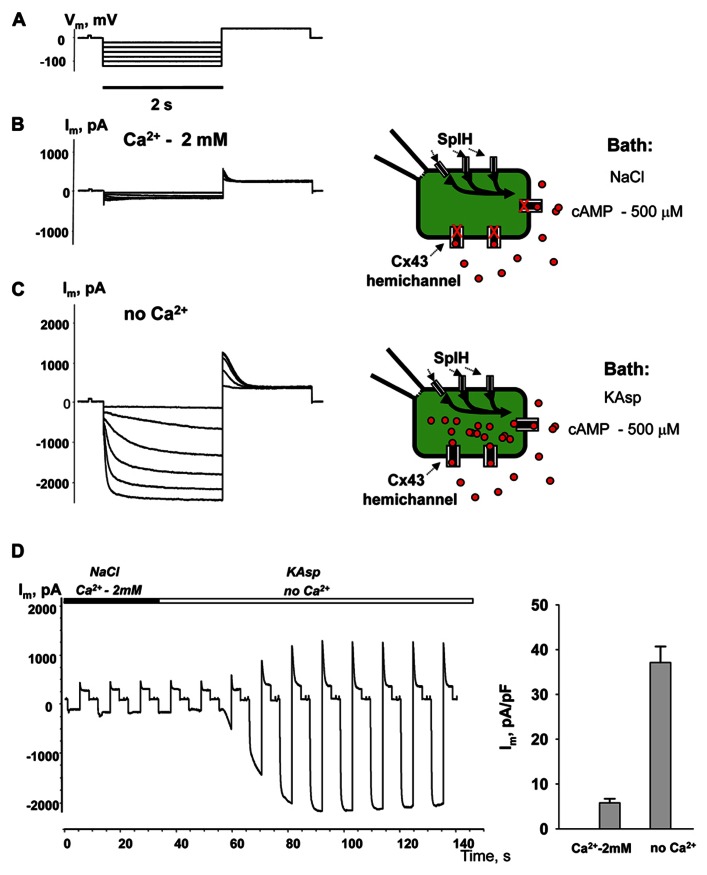
**Detection of intracellular cAMP.** **(A)** Voltage protocol and *I*_m_ recordings from SpIH transfected HeLaCx43 cells perfused with 2 mM extracellular Ca^2^^+^ **(B)**, as well as after the perfusion solution with no Ca^2^^+^ added **(C)** (see schematics on the right for the recording conditions of the cells). **(D)** Current recorded from the HeLaCx43/SpIH cell in response to voltage pulses from a holding potential of 0 to -100 mV, returning to a tail potential of +50 mV. Over time, perfusion with the Ca^2^^+^-free KAsp external solution induced a SpIH current increase to a steady-state. **(E)** Average of current densities measured in the presence of external Ca^2^^+^ and after perfusion with the KAsp solution with no Ca^2^^+^: 5.8 ± 0.9 pA/pF versus 37.1 ± 3.6, *n* = 7, *P* < 0.001, *t*-test. 500 μM cAMP was present in the external solution at any time during the experiment.

**Figure 4** illustrates another series of experiments designed to activate membrane hemichannels by lowering external Ca^2^^+^. In these experiments cells were first perfused with the external NaCl solution containing 2 mM Ca^2^^+^ and 500 μM cAMP (**Figure 4B**). When the NaCl external bath solution was replaced with KAsp bath solution with no Ca^2^^+^, SpIH current increased more than sevenfold (**Figure 4C**). An example of the activation of the SpIH current switching from NaCl bath solution with Ca^2^^+^ to the KAsp solution lacking Ca^2^^+^ is shown in **Figure 4D**. Currents recorded from HeLaCx43 SpIH-expressing cells were derived in response to voltage pulses of -100 mV and returning to a tail potential of +50 mV from a holding potential of 0 mV. At approximately the 30-s time mark, perfusion with Ca^2^^+^-free KAsp solution was initiated. SpIH currents started to increase over time to a new steady-state value due to cAMP diffusion from the external bath into the cytoplasm via membrane hemichannels. On average, derived from seven preparations, switching NaCl bath solution with Ca^2^^+^ to the KAsp solution with no Ca^2^^+^, SpIH currents increased from 5.8 ± 0.9 to 37.1 ± 3.6 pA/pF (**Figure 4E**).

**FIGURE 5:**
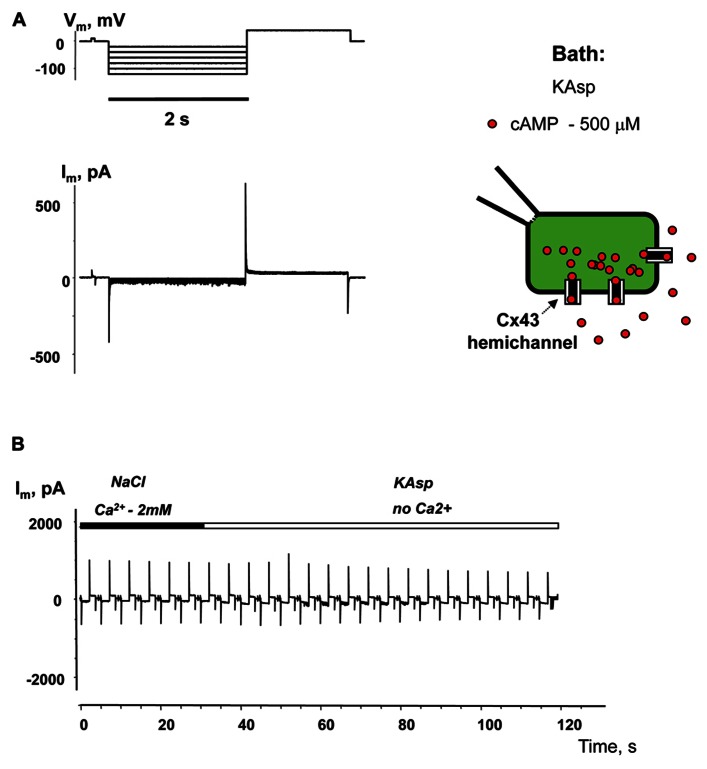
**Membrane currents without SpIH expression.** **(A)** *I*_m_ responses from HeLaCx43 cell lacking expression of SpIH to hyperpolarizing pulses recorded in Ca^2^^+^-free KAsp solution. **(B)** Perfusion with the Ca^2^^+^-free KAsp external solution did not induce currents in HeLaCx43 cells that were not transfected with SpIH (0.4 ± 0.1 pA/pF, *n* = 5).

Experiments performed with HeLaCx43 cells lacking SpIH expression revealed negligible membrane currents in Ca^2^^+^-free KAsp solution with 500 μM of cAMP (**Figure 5A**). **Figure 5B** shows no current increase when the external NaCl bath solution with 2 mM Ca^2^^+^ was exchanged with Ca^2^^+^-free KAsp solution, which is in contrast to the considerable current increase when such a procedure was performed on cells expressing SpIH (**Figure 4D**).

These experiments show that SpIH currents can be activated lowering Ca^2^^+^ in the external solution, because of opening non-junctional hemichannels and following cAMP diffusion into the cell. These observations are consistent with concept that hemichannels activity is regulated by extracellular Ca^2^^+^ (Ebihara et al., 1995;Valiunas and Weingart, 2000;Valiunas, 2002;Contreras et al., 2003b) and HeLa cells expressing Cx43 have been shown to open unopposed Cx43 hemichannels (Contreras et al., 2003a, b).

### VOLTAGE ENHANCED cAMP UPTAKE

**FIGURE 6:**
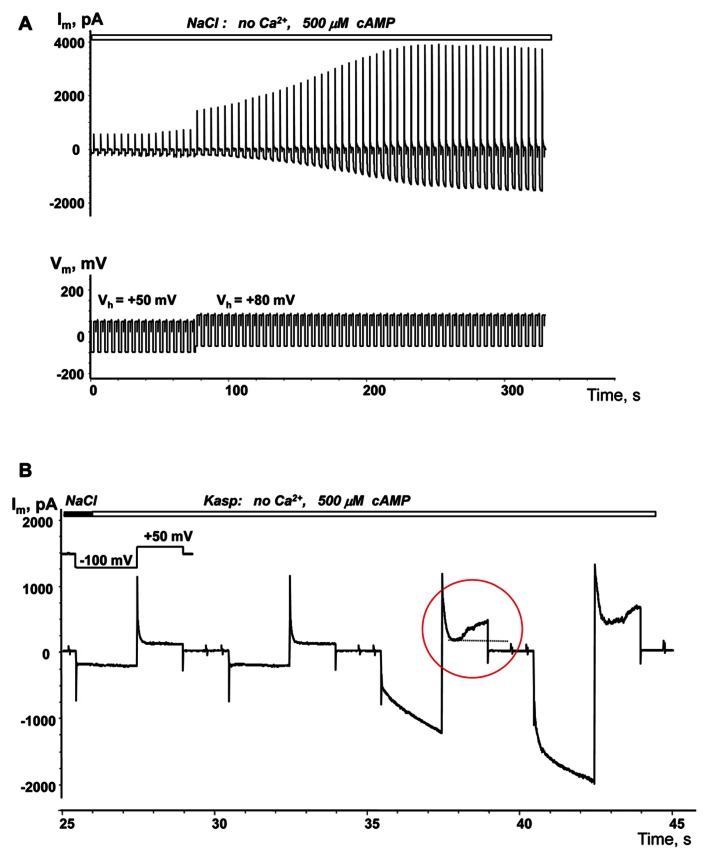
**Depolarization induced cAMP uptake.** **(A)** *I*_m_ recorded from a HeLaCx43/SpIH cell during perfusion with Ca^2^^+^-free NaCl solution containing 500 μM cAMP. Initially the cell was held at *V*_h_ = 0 mV (not shown in the record). There was a weak (~1.3-fold) current increase over approximately 80 s when the cell was held at *V*_h_ = +50 mV. Further cell membrane depolarization to *V*_h_ = +80 mV at ~80 s significantly enhanced the SpIH current in a time dependent manner by ~threefold (when adjusted to the same *V*_h_). **(B)** SpIH current recorded in a HeLaCx43/SpIH cell during perfusion with Ca^2^^+^-free KAsp solution. The SpIH current increase was associated with the time-dependent membrane current increase during depolarization (noted by a red circle), typical of hemichannel activity.

The membrane potential controls hemichannel activity and cell depolarization subsequently triggers the opening of unopposed membrane hemichannels (Ebihara et al., 1995;Valiunas and Weingart, 2000;Valiunas, 2002).An example of depolarization enhanced cAMP uptake is demonstrated in **Figure 6A**. When a single SpIH transfected HeLaCx43 cell was perfused with Ca^2^^+^-free NaCl solution containing 500 μM cAMP there was a very weak SpIH current increase over the 80 s that the cell was held at +50 mV. However, over time, there was a significant SpIH current increase with the resulting saturation when the cell membrane was depolarized and held further at +80 mV (~80 s time mark, **Figure 6A**). Cell depolarization from the resting potential (~-40 mV) to 0 and +50 mV similarly enhanced the SpIH currents in three other preparations. Due to the different holding voltages, the SpIH currents in each cell were analyzed individually. However, in each of the four cells, regardless of the holding potential, depolarization yielded a significant SpIH current increase over the time from 2.2 to 5.1-fold (~fourfold on average in four preparations). In contrast, there was no significant SpIH current increase detected when the cells were held at a hyperpolarizing voltage of -40 mV(6.3 ± 3.8 versus 6.9 ± 3.8 pA/pF, *n* = 3, *P* = 0.7). Not only did lowered extracellular Ca^2^^+^ increase channel activity, but also depolarization caused activation of hemichannels, i.e., more cAMP flux to the cell and subsequent SpIH current increase. An example of a SpIH current increase and associated detectable Cx43 hemichannel activity is demonstrated in **Figure 6B**, where the area circled in red denotes the time-dependent current increase, which implies activation of hemichannels during depolarization.

### cAMP UPTAKE VIA Cx26 HEMICHANNELS

**FIGURE 7:**
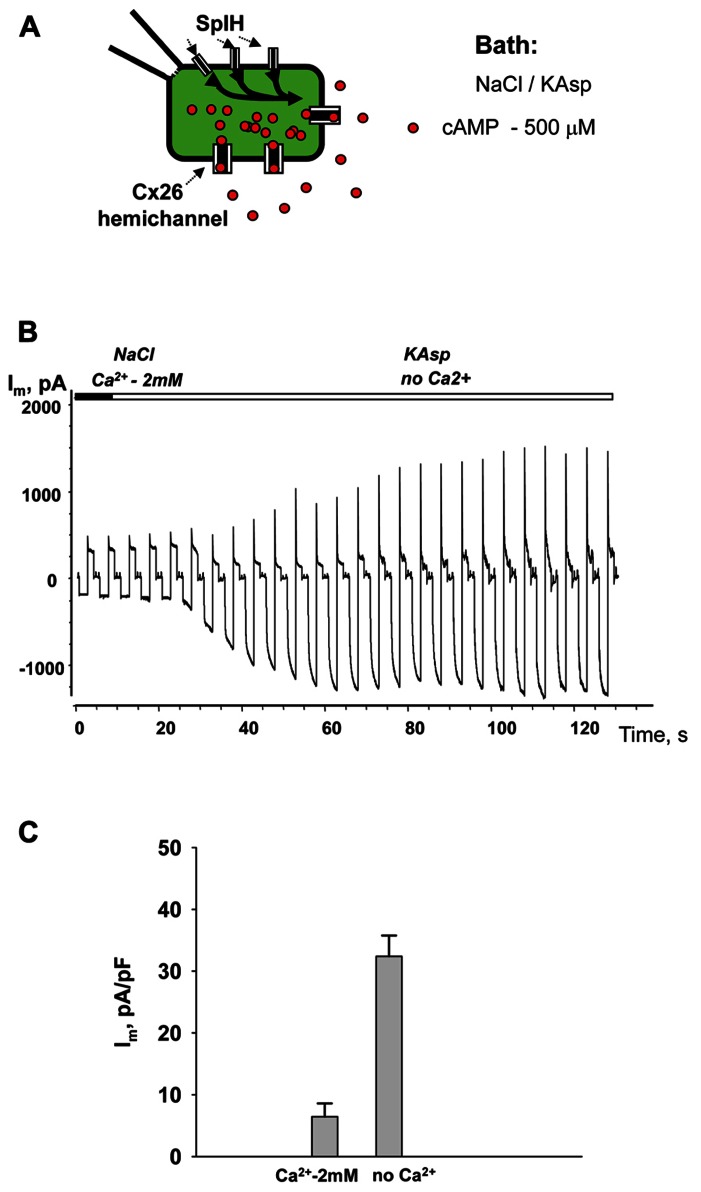
**cAMP uptake via Cx26 hemichannels.** **(A)** Schematic of the experimental environment for HeLaCx26/SpIH cells. **(B)** Current recorded from a HeLa Cx26/SpIH-expressing cell. Perfusion with the Ca^2^^+^-free KAsp external solution induced a significant SpIH current increase over time. **(C)** Average of current densities measured from 5 cells: 6.4 ± 2.2 versus 32.3 ± 3.3 pA/pF, before and after perfusion, respectively; *P* = 0.008. 500 μM of cAMP was present in the external solution throughout the entirety of the experiment.

**FIGURE 8:**
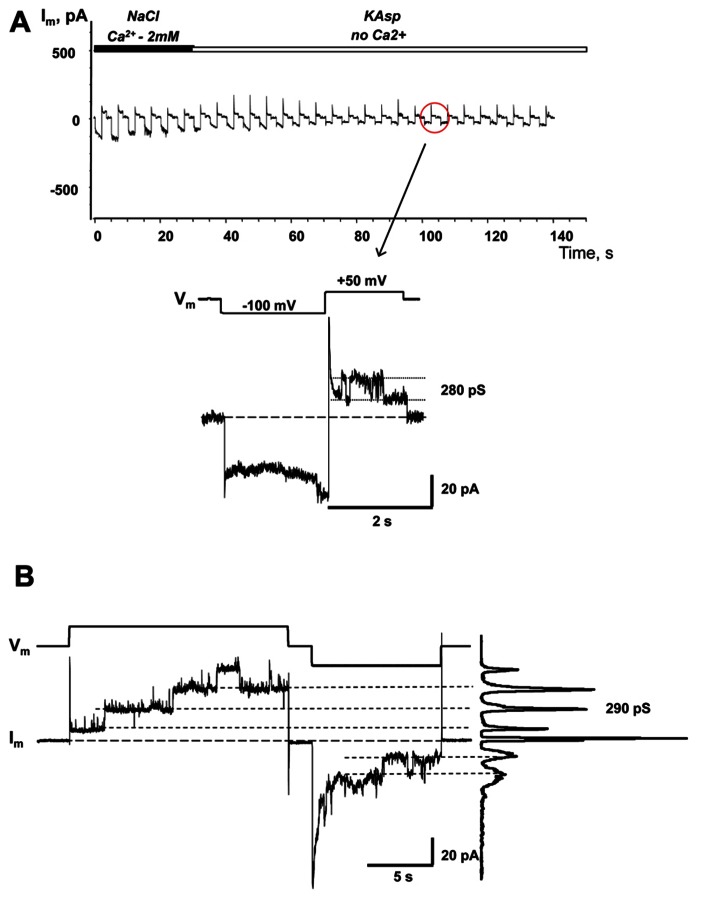
**Single hemichannel currents.** **(A)** Current recorded from a HeLaCx26/SpIH cell. Perfusion with the Ca^2^^+^-free KAsp external solution did not induce a SpIH current increase in four preparations (5.9 ± 1.7 and 6.2 ± 1.9 pA/pF, before and after perfusion, respectively; *P* = 0.886). The operation of single functioning Cx26 hemichannels was visible throughout the experiment. Insert: segment of current recording on expanded time and current scales shows the opening and closing of a Cx26 hemichannel with a unitary conductance of ~280 pS. The external bath contained 500 μM cAMP. **(B)** Hemichannel currents elicited by biphasic 50 mV pulses recorded from HeLa Cx26 cells in a Ca^2^^+^-free KAsp solution. The all point current histograms yielded a conductance of ~290 pS.

Similar to the HeLaCx43 cells shown in **Figures 2–6**, HeLa cells expressing hCx26 also demonstrated cAMP uptake. **Figure 7** shows a schematic of the experimental conditions (panel A) and SpIH current recordings from a HeLaCx26 cell transfected with SpIH (panel B). In five preparations SpIH currents increased from 6.4 ± 2.2 to 32.3 ± 3.3 pA/pF when the NaCl external solution with 2 mM Ca^2^^+^ was replaced with Ca^2^^+^-free KAsp, due to cAMP flux from the external solution through opened membrane hemichannels (**Figure 7C**). In four preparations, some HeLaCx26 cells did not exhibit such SpIH current increases when perfused with Ca^2^^+^-free KAsp solution containing 500 μM cAMP (**Figure 8A**). Such an absence of a current increase could be explained by the lack of the sufficient numbers of operational non-junctional hemichannels in the cell membrane. It has been reported that homotypic Cx26 gap junction channels are~7 times less permeable to cAMP than Cx43 channels (Kanaporis et al., 2008). In the recordings shown here (see insert **Figure 8A**) there are one or two operational hemichannels with ~280 pS unitary conductance detectable in the cell. This suggests that cAMP flux through one or two operational hemichannels is not enough to induce a SpIH current, in contrast to the example in **Figure 7B** where presumably there is a much higher number of open hemichannels in Ca^2^^+^-free solution. Furthermore, phosphodiesterase activity, with such a small number of operational channels, cannot be ruled out completely. Unitary conductance of hemichannels resolved from the record in **Figure 8A**, i.e., ~280 pS, corresponds to unitary conductance of Cx26 hemichannels shown in **Figure 8B**, where the hemichannel current recording was obtained from a HeLaCx26 cell in response to biphasic 50 mV voltage pulses. Discrete steps indicative of the opening (depolarization pulse) and closing (hyperpolarization pulse) of hemichannels are present. The current histograms yielded unitary conductances of 290–300 pS for Cx26 hemichannels, which is in good agreement with Cx26 unitary hemichannel conductance data obtained from *Xenopus* oocytes (Gonzalez et al., 2006;Sanchez et al., 2010).

### LOCAL ACTIVATION OF MEMBRANE HEMICHANNELS

**FIGURE 9:**
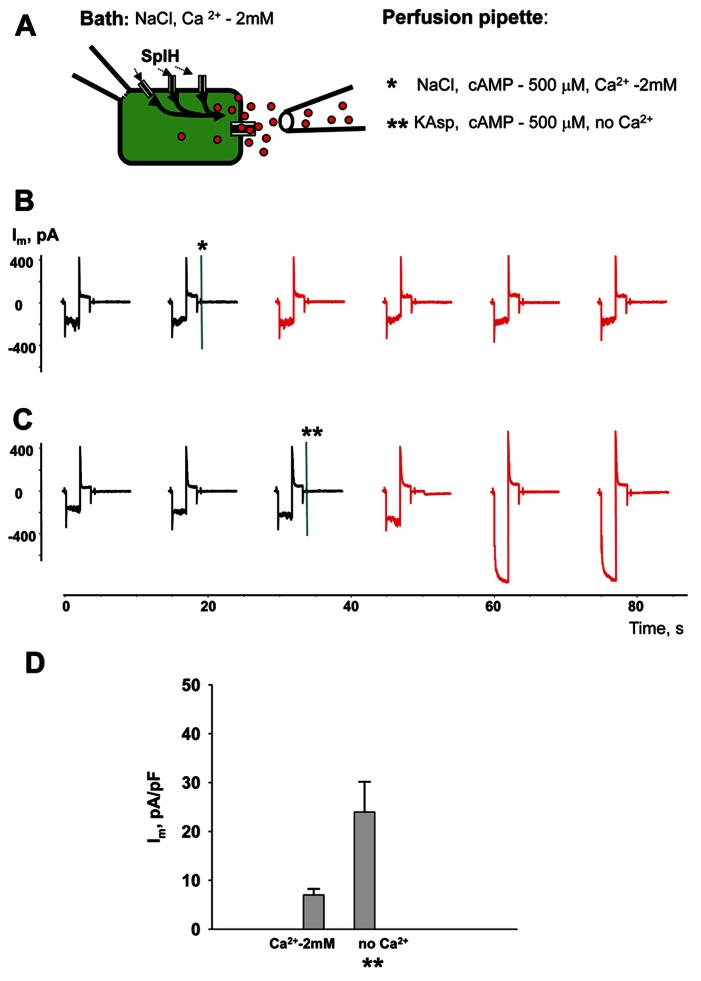
**Local hemichannel activation.** **(A)** Schematic illustrating an experiment with local application of cAMP. The perfusion pipette containing NaCl (*) or KAsp (**) solutions was brought close to the cell and a positive pressure was applied to allow the contents of the pipette to reach the cell membrane. Local application of cAMP via the external pipette did not affect the SpIH current in HeLaCx43/SpIH cells when the perfusion pipette contained the NaCl solution (*):6.6 ± 1.5 and 6.9 ± 1.3 pA/pF, before and during local cAMP application, respectively; *n* = 6, *P* = 0.456 **(B)**. However, the SpIH currents did increase when the pipette contained the KAsp solution (**) **(C)**. **(D)** Summary of current densities: 7.0 ± 1.6 and 23.9 ± 6.2 pA/pF, before and during local cAMP application, respectively; *n* = 6, *P* = 0.041. Currents marked in red in **(B)** and **(C)** correspond to recordings during the local application of cAMP; asterisks (*) and (**) indicate moments when a positive pressure was applied to the perfusion pipette.

Perfusion of the cells with Ca^2^^+^-free KAsp solution activates unopposed hemichannels over the entire surface of the cell membrane. In a series of experiments cAMP was applied locally to a small area of cell membrane through a separate perfusion pipette as illustrated in the **Figure 9A** experimental schematic. HeLaCx43/SpIH cells were bathed in modified Tyrode (NaCl) solution with 2 mM Ca^2^^+^. The currents were recorded with the patch pipette in whole-cell mode. The second perfusion pipette was brought in close proximity to the cell and some positive pressure was applied allowing the contents of the pipette to reach the cell membrane. **Figure 9B** shows the current recording for when the perfusion pipette was filled with NaCl solution containing 2 mM Ca^2^^+^ and 500 μM cAMP. The current amplitude did not change significantly during local perfusion. However, when the perfusion pipette was filled with depolarizing Ca^2^^+^-free KAsp solution and 500 μM cAMP, SpIH currents increased significantly, indicating a cAMP influx through open hemichannels (**Figure 9C**). **Figure 9D** shows averaged current amplitudes recorded before and during local cAMP application.

**FIGURE 10:**
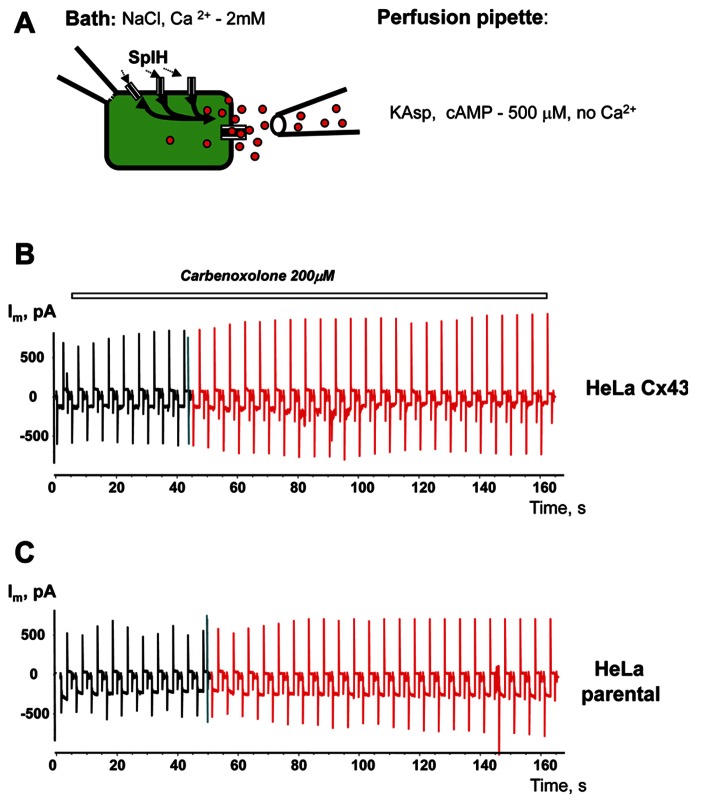
**Modulation of hemichannel activity.** **(A)** Schematic of the experiment. **(B)** SpIH current recordings from a HeLaCx43/SpIH cell perfused with Ca^2^^+^-free KAsp external solution containing 200 μM carbenoxolone. No SpIH current increase was detected during local application of cAMP (red current traces) in five cell preparations (6.8 ± 2.2 and 6.6 ± 2.8 pA/pF, before and during local application of cAMP, respectively; *P* = 1.0). **(C)** SpIH current recorded from connexin deficient HeLa parental cells transfected with SpIH during local application of cAMP (red current traces; 6.8 ± 1.9 and 8.7 ± 2.8 pA/pF, before and during local application of cAMP, respectively; *n* = 10, *P* = 0.734.

To prove that cAMP flux is via hemichannels, similar experiments were performed with the presence of the gap junction channel blocker carbenoxolone (200 μM) in the external bath solution. Local perfusion (**Figure 10A**) with depolarizing KAsp solution (500 μM cAMP, Ca^2^^+^-free) did not induce a SpIH current rise in HeLaCx43/SpIH cells (**Figure 10B**). Connexin deficient HeLa parental cells transfected with SpIH likewise did not exhibit a SpIH current increase during local perfusion with cAMP (**Figure 10C**).

These experiments show that membrane hemichannels can be activated over the small area of the cell membrane and allow passage of cAMP into the cell.

## DISCUSSION

The data presented here show that hemichannels or connexons of Cx43 and Cx26 are permeable to cAMP as demonstrated by the cAMP sensor SpIH. The data address one essential question: are hemichannels to be considered an alternate pathway for autocrine and paracrine delivery? That is, hemichannels are permeable to one signaling molecule known to be involved in paracrine functions utilizing the extracellular pathway (Dahl and Locovei, 2006;Anselmi et al., 2008;Kanaporis et al., 2008;Kang et al., 2008;Wang et al., 2013).

One issue that has yet to be clearly addressed is the true operational limits of hemichannels. Previous works have often used lowered extracellular Ca^2^^+^ to allow easy demonstration of hemichannel currents. When extracellular Ca^2^^+^ is in the μM range, macroscopic or multichannel data has revealed that hemichannels have a very high open probability with depolarization (Ebihara et al., 1995;Valiunas and Weingart, 2000;Beahm and Hall, 2002;Valiunas, 2002;Gonzalez et al., 2006).

Ischemia is known to result in membrane depolarization of cardiac myocytes (Kleber et al., 1978;Kleber, 1983) and cells of other aerobic tissues (Al-Mehdi et al., 1998;Calabresi et al., 1999;Dreier, 2011). As such, hemichannels whether activated by altered Ca^2^^+^ levels and/or membrane depolarization have the potential to allow both the influx and efflux of solutes. Consistent with this notion is activation of hCx26 hemichannels at voltages above -40 mV (Steffens et al., 2008). In the case of ischemia it is not yet clear whether hemichannel activity is the cause of membrane depolarization or is simply part of the effect. One can speculate that if hemichannels are causal in ischemic depolarization then hemichannels become real therapeutic targets.

The large conductance and permissive selectivity of Cx43 and Cx26 would also allow significant delivery of solutes like cAMP, possibly with even extremely low open probabilities on the order of 0.1–1%. In fact, little is known about the activity of hemichannels when extracellular Ca^2^^+^ is between 1 and 2 mM, the expected range for the interstitial space. What remains to be determined is the open probability of hemichannels versus extracellular Ca^2^^+^ while utilizing conditions to silence other channel types, i.e., K^+^ channels. For a cell with 10000 hemichannels on its surface and with an imaged open probability of 1% in normal extracellular Ca^2^^+^ the number of functioning channels would be approximately 100 open channels at any instant in time. Assuming the cAMP/K^+^ permeability ratio for Cx43 and Cx26 hemichannels is similar to their respective gap junction channels then the flux per channel is as previously reported (Kanaporis et al., 2008), approximately 6000 molecules/channel/sec for Cx43 and approximately 1800 molecules/channel/sec for Cx26. For 100 functioning channels the total flux per cell would be 0.6 million molecules/sec for Cx43 and 0.18 million for Cx26. Whether such an efflux is sufficient to function as an autocrine/paracrine source of signal molecules like cAMP remains to be seen.

Understanding the potential role of hemichannels, unrelated to their precursor role in the formation of gap junction channels, as a potential autocrine/paracrine signaling pathway is a real challenge. It is challenging both from a biophysical perspective and the assessment of autocrine and paracrine effects within tissues. Open probability versus calcium is an important biophysical parameter to clearly define, but it is also necessary to test autocrine/paracrine delivery mediated by hemichannels without an endosomal/vesicular background. The latter can be accomplished using drugs that inhibit or block endosomal/vesicular traffic. This study focused on hemichannels composed of connexins but an equally plausible hemichannel construct is a hemichannel composed of pannexins (Wang et al., 2013). Thus, it remains to be seen whether connexins or pannexins are truly able to function as autocrine/paracrine-like delivery systems.

In this study, the SpIH gene, which is a cyclic nucleotide gated channel, was used to assess the permeability of hemichannels to cAMP. This is a useful method, which allows accurate estimates of cyclic nucleotide flux via different membrane hemichannels. In such cases, it is the most suitable approach for quick screening of connexin mutants. Defining the permeability and selectivity properties of hemichannels are important factors in understanding their potential role in normal cell physiology and disease states with connexin mutations.

A final question posed is if there is a role for autocrine and paracrine delivery mediated by hemichannels within the myocardium? Presently autocrine and paracrine functions for hemichannels are open to debate, but there is strong evidence that cAMP influx can act to reduce the sodium current in cardiac myocytes (Hofer and Lefkimmiatis, 2007). Autocrine and paracrine-like functions are also a possible explanation for enhanced endothelin expression in response to ischemia or hypertrophy (Drawnel et al., 2013).****

A number of reports reviewed inWang et al. (2013) demonstrated that hemichannels can function as pathways for paracrine messengers, including ATP and prostaglandins. This study now adds an additional potential paracrine-like messenger in the form of cAMP.

## Conflict of Interest Statement

The author declares that the research was conducted in the absence of any commercial or financial relationships that could be construed as a potential conflict of interest.
